# The First Telementoring Programme in Latvia: A Qualitative Study of the “ECHO School of Psychiatry” for General Practitioners

**DOI:** 10.3390/healthcare14081044

**Published:** 2026-04-15

**Authors:** Marija Burceva, Vineta Viktorija Vinogradova, Elmars Rancans

**Affiliations:** Department of Psychiatry and Narcology, Riga Stradins University, Tvaika Street 2, LV-1005 Riga, Latvia; vineta.vinogradova@rsu.lv (V.V.V.); elmars.rancans@rsu.lv (E.R.)

**Keywords:** primary health care, psychiatry, continuing education, general practitioners, telementoring, ECHO project

## Abstract

**Background/Objectives**: Previous research has shown that mental disorders are common in the general population in Latvia, while access to specialised psychiatric services is limited, particularly in rural areas. General practitioners, therefore, have a crucial role in the early detection and management of these conditions. Previous studies and national initiatives have highlighted an unmet need for continuing education in psychiatry tailored to the Latvian primary care context. In response, the first Latvian telementoring programme, the “ECHO School of Psychiatry” (Extension for Community Healthcare Outcomes, ECHO), was launched in 2023 to enhance general practitioners’ competencies and decision-making in mental healthcare. This study explored general practitioners’ experiences and perceptions of participation in the programme and its perceived impact on their practice, using a qualitative approach. **Methods**: Thirteen women general practitioners who had participated in the programme between October 2023 and February 2025 were recruited using voluntary response sampling, via email invitations from programme coordinators. Individual semi-structured interviews were conducted remotely between May and September 2025, audio-recorded, transcribed verbatim, and the resulting transcripts were analysed thematically using an inductive approach, supported by NVivo software. Data collection continued until no new themes emerged. **Results**: Four main themes emerged from the thematic analysis: (1) participants’ perceptions of the structure and educational value of the programme; (2) perceived impact of the programme on clinical practice and decision-making; (3) programme limitations in addressing professional isolation and fostering collaboration; (4) suggestions for programme improvement. Themes illustrate participants’ perceptions of the programme’s value, its impact on practice, and recommendations for further development. **Conclusions**: This study provides insights into the strengths and areas for improvement of the “ECHO School of Psychiatry” as perceived by general practitioners. It also acknowledges current challenges in primary care, such as limited access to specialists and professional isolation.

## 1. Introduction

Mental disorders are common globally, with the prevalence rising notably in recent years [1]. A recent study conducted in Latvia revealed a prevalence of 37.2% for at least one mental disorder in the primary care population [2]. The Latvian healthcare system is facing a significant shortage of doctors in various fields, including mental health, with rural regions being disproportionately affected [3]. Considering the limited availability of mental health care in rural areas, the knowledge and skills of primary care physicians in diagnosing and managing psychiatric conditions are particularly important [4,5,6]. In Latvia, medical education comprises six years of undergraduate training, followed by four to five years of specialised residency. While this model provides foundational knowledge, it may be limited in its ability to develop specific skills in the management of mental disorders in routine primary care practice.

Several studies have highlighted that mental disorders are a prevalent diagnostic category in primary care and represent particular challenges, including diagnostic uncertainty, frequent comorbidity, and time constraints for comprehensive patient assessment [7,8]. Moreover, general practitioners have a pronounced need for structured and extensive training to strengthen their competencies and capacity to provide mental healthcare [8].

In 2016, a targeted training module for general practitioners was developed. Under this module, ten “Depression School” seminars were held in several regions of Latvia to improve the recognition and management of depression in primary care [7]. Evaluation of this intervention demonstrated a statistically significant and sustained improvement in depression diagnostic practices among participating doctors following training [9]. Before this initiative, general practitioners had not received targeted continuing education programmes in psychiatry. This intervention also explored general practitioners’ experiences in the management of mental disorders, revealing considerable challenges, such as diagnostic uncertainty, complexity of cases, and time constraints [7]. Furthermore, it emphasised the necessity for the enhancement of knowledge within a broader thematic scope [7,8].

Subsequently, a cross-sectional study conducted in 2023 involving 150 general practitioners nationwide further confirmed this unmet need, additionally identifying diagnostic categories perceived to be the most complex: affective, somatoform, and neurotic disorders [10]. These findings emphasise the importance of encompassing a broader spectrum of conditions within continuing education initiatives, thereby enhancing their diversity, sustainability, and alignment with the specific requirements of Latvian primary healthcare.

One of the widely used and practical approaches to disseminating and strengthening specific skills and knowledge among healthcare professionals is the implementation of telementoring programmes [11]. These involve expert clinicians providing guidance to less experienced practitioners via remote technology, helping to overcome geographical barriers to education and collaboration [12]. The ECHO (Extension for Community Healthcare Outcomes) project is a widely implemented telementoring model that facilitates the transfer of knowledge and best practices across medical specialties [13,14].

The ECHO model was developed in New Mexico, USA, in 2003 and has since encompassed over 8700 programmes across 214 countries and areas [15]. The model also facilitates mutual learning between experts and regional clinicians, reflecting the core principle of ECHO—“all teach, all learn” [15].

In Latvia, the first ECHO initiative, the “ECHO School of Psychiatry”, was launched in 2023 by the Department of Psychiatry and Narcology of Riga Stradins University. The primary objective of this project is to improve the competence of general practitioners and strategic decision-making in the treatment of patients with mental disorders. Despite the evidence of the effective implementation of the ECHO model on a global scale, its adaptation and applicability within the specific national healthcare system context in Latvia remains unexplored.

To evaluate the effectiveness and impact of this first ECHO telementoring programme in Latvia, a systematic research process has been initiated, encompassing a variety of research methods. This study focuses on exploring participants’ experiences and perceptions of the programme using a qualitative approach. A review of research conducted on ECHO programmes worldwide reveals the employment of both quantitative and qualitative methods. The latter approach has been shown to offer unique insights into subjective experiences and emotional aspects, whilst also enabling the interpretation of complex social phenomena that cannot be accessed through quantitative methods alone [16].

This study was conducted to preliminarily evaluate the project “ECHO School of Psychiatry” as the first telementoring initiative in Latvia, from the perspective of participating general practitioners. Using qualitative methods, the assessment focused on perceived usefulness and relevance of the programme, as well as its potential to address the identified mental health education gap within primary care. These insights are essential for guiding further development and optimisation of the programme, offering an understanding of how participants experienced the programme and its perceived relevance for their clinical practice.

## 2. Materials and Methods

The study was approved by the Ethics Committee of Riga Stradins University (No. 4/611/2025 from 27 March 2025).

### 2.1. Intervention and Study Design

This qualitative study employed individual semi-structured interviews with general practitioners following their participation in the “ECHO School of Psychiatry”.

The “ECHO School of Psychiatry” operates as a telementoring programme comprising eight online seminars attended by groups of 25–50 general practitioners from various regions of Latvia. The programme aims to enhance general practitioners’ knowledge and strengthen their clinical confidence in managing mental disorders. Seminars are designed to promote active interaction among participants as well as between participants and psychiatrists, focusing on the analysis of real challenges encountered in practice. Each session includes a didactic lecture and case-based discussions, offering participants the opportunity to present and analyse their own challenging clinical cases. Each of the eight sessions focuses on a specific diagnostic group or aspect of mental healthcare relevant to primary care (Table 1). These diagnostic categories were based on the ICD-10 (International Classification of Diseases, 10th Revision) classification system used in Latvia and were selected to reflect mental health conditions and symptom presentations that are commonly assessed and initially managed by general practitioners in primary care. The programme also provides access to session recordings, presentations, and supplementary educational materials to support learning beyond the programme. To date, three training groups have been conducted, with 108 general practitioners enrolled in the programme and 93 having completed it.

This qualitative study was grounded in a relativist ontology and a constructivist epistemology, acknowledging that meanings are shaped through subjective experience. This particular stance was selected as appropriate for exploring general practitioners’ perspectives, attitudes towards the programme, and its perceived impact on their clinical practice.

The study was designed and reported in accordance with the 32-item Consolidated Criteria for Reporting Qualitative Health Research (COREQ).

Thematic analysis by Braun and Clarke (2006) was employed to explore patterns within participants’ narratives and identify key themes and concepts [17].

### 2.2. Participants

The study sample comprised all three groups of general practitioners (n = 108, 93.5% female) who participated in the “ECHO School of Psychiatry” programme between October 2023 and February 2025. A voluntary response sampling method was used to recruit participants.

Personalised email invitations to participate in the study were sent to all general practitioners enrolled in the programme across its three groups. Interested doctors were provided with comprehensive information about the study, and written consent was obtained before their enrolment.

Of the 108 eligible general practitioners enrolled in the programme, including those who discontinued participation before completion, 13 were interested and agreed to participate in the interviews: four participants from the first group, five from the second, and four from the third. Information on reasons for non-participation was not systematically collected; therefore, the perspectives of general practitioners who chose not to participate, including those who may have had less satisfactory experiences, may not be represented.

### 2.3. Interview Topic Guide

A topic guide was developed (Table 2) to explore general practitioners’ perceptions of the “ECHO School of Psychiatry”, its relevance to primary care challenges in Latvia and perceived impact. The topic guide was refined through an iterative process, with initial interviews informing the addition of follow-up prompts to support participants in recalling specific programme aspects when needed, which helped sharpen the focus of subsequent interviews on relevant areas identified in the initial discussions. These prompts served only as guidance, preserving comparability across interviews.

### 2.4. Data Collection

Data collection was conducted by the first author (MB), a researcher who was involved in coordination but had no teaching role in the programme, through individual semi-structured interviews between May and September 2025. All interviews were conducted remotely via Zoom at a time chosen by each participant, using their preferred device. No other individuals were present during the sessions.

The average interview duration was 14 min (range: 9–21 min). All sessions were audio recorded and transcribed verbatim using Microsoft 365 software, with permission obtained from all participants. The transcripts were not returned to participants for review and correction, and no repeat interviews were conducted due to participants’ limited availability, which is acknowledged as a methodological limitation.

Data collection continued until data saturation appeared to be reached, as agreed by all members of the research team. Saturation was defined as the point at which no novel explanations, ideas, and codes emerged from additional interviews. In this study, data saturation was observed after the 11th interview, when no new codes were identified. Two additional confirmatory interviews were conducted, which confirmed the stability of the codebook and themes, bringing the total number of interviews to 13. Nonetheless, the relatively brief duration of the interviews may have limited the depth and richness of the data and the capacity to explore more complex themes; accordingly, the findings should be interpreted with due caution.

### 2.5. Data Analysis

Audio recordings were transcribed using Microsoft 365 software, followed by systematic review and editing. All transcripts underwent multiple review cycles before analysis.

Thematic analysis by Braun and Clarke (2006) [17] was applied to the data. Familiarisation was achieved through repeated reading of the transcripts accompanied by analytic note-taking. Initial coding was conducted by M.B. and subsequently reviewed by V.V.V., with recommendations for refinements to the coding scheme. Coding and data management were performed using NVivo 12 software.

Preliminary themes were developed from the data using a primarily inductive approach, informed by the research questions and the interview guide’s focus. Subsequently, the research team reviewed these themes collaboratively until consensus was reached. They were examined in relation to the complete data set and final theme definitions, and descriptions were formulated.

To enhance credibility and rigour of the analysis, coding and theme development were conducted iteratively, with the research team members holding discussions to review coding decisions and refine theme boundaries. Coding disagreements were discussed until consensus was reached. An audit trail was maintained to document coding decisions, code revisions, and theme development. Given that members of the research team were involved in the implementation of the programme, particular attention was paid to reflexivity. The potential impact of the interviewer–participant relationship was identified, and its possible influence on social acceptance bias was assessed. Reflective notes covered not only coding decisions, but also documented researchers’ positionality and its potential impact on data interpretation, supporting critical evaluation and minimisation of confirmation bias. The research team reviewed the data iteratively to ensure that themes accurately reflected participants’ accounts and that divergent or less frequent perspectives were not overlooked.

## 3. Results

### 3.1. Results of the Thematic Analysis

A total of 33 final codes were generated during the coding process, supported by 152 quotations. The codes were organised into 14 subthemes, with 4 main themes emerging across the dataset (Table 3). Collectively, the themes illustrate participants’ perceptions of the programme’s relevance and value, its impact on clinical practice, and their comprehensive suggestions for further improvement. Illustrative quotes were selected to reflect the main themes and to provide contextual insight into the participants’ experiences, supporting the interpretation of the results.

### 3.2. Participants’ Perceptions of the Structure and Educational Value of the Programme

Participants reported positive overall impressions of the “ECHO School of Psychiatry”, emphasising its structured format, practical orientation, and the relevance of its topics. Most participants described it as well-suited to the needs of Latvian general practitioners because of its accessibility, practical orientation, and direct relevance to current clinical challenges. Several participants framed the programme within a broader perceived lack of structured continuing education in psychiatry, particularly in settings with the limited availability of specialised care. Participants tended to describe it as a partial response to these constraints.

*“The “ECHO School of Psychiatry” was a great opportunity to go through the entire cycle of psychiatry and put it all in order: to remember forgotten things, learn new things, and feel more confident with this group of diseases.”*

*(Participant 12)*

Participants emphasised the interconnection of the programme’s thematic scope and telementoring format in shaping its perceived value. They noted its systematic focus on the most challenging diagnostic categories and clinical situations highly relevant to primary care. The structured sequence of sessions was described as enabling a broader overview of everyday clinical challenges. This indicates that participants valued the coherent sequencing as supporting a more organised understanding of topics.

*“I am actually very excited that such a project is being organised at all. One thing is to attend a seminar or lecture, but on a specific topic or part of it. In this case, when a whole lecture course or a series of classes is put together, we have the opportunity to take a closer look at what we actually work with daily. And that is great.”*

*(Participant 4)*

The accompanying materials were perceived as valuable tools that could be integrated into practice. Participants characterised these resources as extending the educational impact into their routine clinical work. This reflects that participants integrated the materials into their own practice, enhancing the practical utility of the programme.

*“I saved the tables with medications and guidance on which to use in any case, and diagnostic algorithms… I opened them, looked at them alongside the sessions, and saved them. They have created a kind of personal library for me to use when I need it.”*

*(Participant 10)*

While most participants valued the structured thematic scope and additional materials provided, one participant expressed dissatisfaction with the excessive focus on guidelines and classifications, highlighting their insufficient alignment with the realities of clinical practice and the decision-making processes within it.

*“Classifications and guidelines do not really help me. In practice, they often do not work because I personally do not have the time to engage deeply with them. How do endless classifications help you in your daily work? They do not… It is just theory. But what should you actually do in real life—which diagnosis should you choose, which medication should you prescribe?”*

*(Participant 7)*

This contrasting perspective highlights that participants’ perceived value of the programme depended not only on structured materials, but also on the opportunities to apply knowledge in real cases, which the programme sought to support through the telementoring format and case-based discussions. This illustrates that participants’ perceived value of the programme depended on the applicability of the content to their clinical decisions.

The telementoring format was valued for its convenience and suitability, facilitating participation by overcoming geographical barriers and reducing time constraints. Participants also highlighted the availability of seminar recordings and materials, which they regarded as important for supporting continuous learning beyond the formal programme duration, particularly in the context of high workload.

*“Well, for me it is almost 150 km to Riga, so I definitely preferred the remote format. Yes, because you can drive to a seminar once, but that regular travelling—well, that’s… not really feasible.”*

*(Participant 9)*

*“I also liked the fact that the recordings remained available for some time… There were both the recordings and the materials. And if I joined only partially… I could watch it later as a recording.”*

*(Participant 10)*

The case-based discussions were identified as a helpful tool for supporting participants’ development of clinical competencies, particularly when combined with guidance from psychiatry experts. These discussions enabled participants to reflect on their own cases, gain insights from peers, and receive targeted advice from specialists.

*“When specific clinical cases are analysed, we can often recognise our own patients in these examples. This allows us to evaluate how we have acted, and how the specialist, who may work daily with patients in this field, has managed the case. It also helps us understand that we can manage much of it ourselves, given the limited availability of specialists. I particularly appreciate this content because it is not just theory; it involves cases with real patients, including their anamneses and related data.”*

*(Participant 1)*

*“Participants themselves presented clinical situations from their practice. And then the specialist gave practical advice. It was very valuable to see the experiences of other general practitioners and the specialists’ views on them. In that sense, it was a good balance.”*

*(Participant 12)*

The safe learning environment created by the psychiatry specialists was frequently mentioned, encouraging participants to ask questions freely and allowing them to receive practical tips beyond the scheduled topics.

*“What I liked the most was the personal communication with the psychiatry specialists, the opportunity to truly ask all the questions I was interested in… I felt completely comfortable. They were friendly and supportive… And the lecturers were always accommodating, no matter how silly a question might have sounded.”*

*(Participant 5)*

These supportive elements, including the format, thematic scope, case-based discussions, and expert guidance, were perceived by participants as helpful in applying knowledge in practice and influencing their clinical decision-making. These findings suggest that the perceived value of the programme is closely linked not only to its content but also to its delivery format, which reduces structural barriers to participation commonly experienced by general practitioners.

### 3.3. Perceived Impact of the Programme on Clinical Practice and Decision-Making

Participants perceived the “ECHO School of Psychiatry” as impacting their confidence in clinical knowledge and decision-making. According to participants, this occurred either by confirming the appropriateness of existing management strategies or by providing additional knowledge supporting their continued practice.

*“I really appreciated the opportunity to feel confident that a general practitioner can also manage the treatment of patients with mental disorders… I have started to feel much more confident working with patients with mental health disorders overall, not only with depression.”*

*(Participant 9)*

*“Yes, what I liked was the confirmation that I seem to be doing everything correctly. I gained reassurance regarding my own practice.”*

*(Participant 11)*

For some participants, the programme helped clarify the boundaries of what can be managed independently in primary care, recognising situations requiring referral to a psychiatrist.

*“And it was reassuring for me… I realised that I do not have to know everything; it is enough to know these few things I do… I can initiate treatment and refer a patient to a psychiatrist. Well, it was comforting to know that I have support from professionals and that I do not have to try to fight on my own… It also gave me the understanding of how much we can manage in primary care, and where specialised care becomes necessary.”*

*(Participant 4)*

*“I am more confident in prescribing antidepressants… I have clearer boundaries—when it is my patient and when it is a case for a psychiatrist…”*

*(Participant 2)*

Participants reported that the programme influenced their daily clinical practice, particularly in ways they perceived as supporting their understanding of patients and their concerns, and guiding clinical decisions. It is important to mention that these accounts reflect participants’ self-reported experiences rather than objectively measured changes in patient care.

*“I have successfully prescribed antidepressants to several patients… Some patients have given me feedback that it has helped very, very well. These were very successful cases… Patient’s quality of life, health, feelings and emotional well-being improved significantly.”*

*(Participant 2)*

For example, several participants described more frequent use of the screening instruments, mainly the Patient Health Questionnaire-9 (PHQ-9) and Generalised Anxiety Disorder-7 (GAD-7), which they noted helped them to identify patients with mental health-related symptoms early and supported more informed decision-making.

*“For example, it is much easier now: I use those questionnaires more. And those people with particular problems really do get identified… Somehow, you notice those people more. You look at them differently.”*

*(Participant 3)*

*“Well, I approach people more. I used to avoid doing that. But now I genuinely try to ask people more about how they feel and give them the questionnaire to complete. And I see that people like it and find it useful. Overall, I find it is useful as well.”*

*(Participant 6)*

### 3.4. Programme Limitations in Addressing Professional Isolation and Fostering Collaboration

Although participants reported various positive aspects and effects of the “ECHO School of Psychiatry”, the programme was perceived as not fully addressing the professional isolation that might be experienced by general practitioners. This limitation was attributed to limited engagement from other participants and the constraints of the remote format.

*“In Latvia, the mentality is not such that we have wide discussions. Usually, you have to wait a long time for an answer, because I think about what I will say… We are generally shy to ask questions. As a result, the lecturer’s feedback may be limited. Even among 50 people, we do not talk much, we do not share. Everyone tends to hold back a little.”*

*(Participant 2)*

*“Quite a lot of those who participated did not have cameras, and well, it felt a bit strange at times… There were a couple of questions that I did not ask myself, because I felt I was not really free to ask a few times… Maybe it was because I could not see all the other people’s faces.”*

*(Participant 8)*

Participants also described how the remote format itself influenced interaction, with technology acting as a mediator, limiting spontaneous exchange and mutual engagement.

*“It would have been nicer if the audience had been more responsive… It isn’t easy to overcome that threshold—to communicate through technology… And then there was somewhat of a lack of mutual interaction that might have been present in person.”*

*(Participant 6)*

The findings suggest that, despite the intended “community of practice” model, social and cultural factors may limit the development of collaborative learning environments.

### 3.5. Suggestions for Programme Improvement

Despite the overall positive evaluation of the “ECHO School of Psychiatry” and its perceived impact on daily clinical practice, participants also identified several areas for improvement to tailor the programme to the specific needs and realities of Latvian general practitioners.

Participants expressed a need to expand the programme’s thematic scope and include additional diagnostic categories that are commonly encountered and perceived as challenging, with specific emphasis on topics related to child and adolescent psychiatry.

*“Perhaps borderline personality disorder and schizophrenia could also be included, and the ability to recognise them, as this is essential in practice.”*

*(Participant 11)*

*“… I would need to cover organic disorders, which we actually observe in many seniors in practice.”*

*(Participant 4)*

*“Also, you could add more about children… I have been working since the 90s, and there are so many children with autism spectrum disorders. I think that in the last five years I have seen more than in the previous 30 years combined… I think there could be an entire lecture cycle on this. Well, it is a broad field with a lot of room to expand into.”*

*(Participant 6)*

Several participants suggested extending the programme beyond psychiatry to other medical fields, noting the potential usefulness of the ECHO telementoring model for continuing education.

*“Definitely do not just stick to psychiatry, but perhaps expand! Go a little bit further into other topics: paediatrics, neurology, infectious diseases. Well, all of these would also be very, very desirable.”*

*(Participant 1)*

*“I would be delighted if remote programmes were also available in other fields… There is also dialogue there. There are all the opportunities to ask questions and meet specialists to consult.”*

*(Participant 9)*

The incorporation of supplementary educational materials and practical tools was recommended to support knowledge retention and clinical decision-making. Participants suggested concise summaries and step-by-step algorithms that could be adapted to each patient’s circumstances.

*“… For example, when a topic or the whole lecture is finished, there could be a single slide summarising everything, where the lecturer also mentions the main points to remember. It would serve as a reinforcement, because later, when you glance at that summary, everything about that lecture, about that topic, comes back to mind. Such key points in a single file or slide would be useful for better retention.”*

*(Participant 10)*

*“We need practical algorithms and recommendations. I would even say: step-by-step algorithms, which would, of course, be adaptable to a more individual situation. With steps marked as “yes” or “no”, indicating certain actions we take, for how many weeks we do them this way. That would be the ideal scenario.”*

*(Participant 12)*

Participants also suggested measures to support active learning and foster more active engagement of doctors in the programme. This included interactive activities such as polls, organisational adjustments such as formation of smaller groups, and encouragement of participants to submit clinical cases. These strategies were perceived as likely to increase participation, promote more open discussions, and enhance reflection.

*“Instead of just reading theory, put it to a poll and ask general practitioners themselves the question, “What do you think?”. So that we can learn together, ask questions and give answers… And then look at the percentages of who answered what, and evaluate our responses… Make it more interactive… Because I think it is very memorable for people.”*

*(Participant 8)*

*“It really should be like that: during the cycle everyone should be required to submit at least one clinical case… That would also encourage more mutual communication.”*

*(Participant 6)*

*“Personally, I think that 15–20 would be the optimal number of participants… Of course, in a smaller group, I think people are also more open.”*

*(Participant 8)*

Finally, the participants recommended repeating the programme for past participants to continuously update knowledge and support the development of competencies in light of ongoing developments in psychiatry. Expansion of the programme was recommended to reach a wider audience, reflecting the perceived value of the “ECHO School of Psychiatry” not only for general practitioners but also for other medical specialities, potentially supporting patient care competencies across disciplines and enabling the provision of more thoughtful, informed care to individuals with mental disorders.

*“… In medicine, everything is changing and moving rapidly. Therefore, if there are any current updates, new guidelines, new classifications, new medications, these are certainly worth including, expanding on, and repeating. I would participate again!”*

*(Participant 10)*

*“I think this is useful for any specialist—a neurologist, a cardiologist… I think any specialist in any field should pay attention to mental disorders to identify and address these symptoms early, so that it becomes much easier for doctors to manage patients’ physical conditions.”*

*(Participant 11)*

*“Maybe after some time… It would be possible to organise another meeting for the groups that have already completed the training. Yes, to see how they are doing, to give some interesting lectures again. So, you have already tried something in practice, and then you have new questions…”*

*(Participant 5)*

Collectively, these suggestions underscore participants’ focus on expanding the programme’s thematic scope, integrating practical tools, and fostering interactive engagement, indicating that participants valued the programme for its applicability and usability in daily clinical practice. These suggestions indicate a demand for more structured, practice-oriented, and interactive learning formats, reflecting the need for continuous support rather than one-time educational interventions.

## 4. Discussion

This qualitative study explored general practitioners’ perceptions of Latvia’s first telementoring programme, the “ECHO School of Psychiatry”, identifying its perceived strengths and areas for further development.

Participants’ reflections were situated within a broader context of the rising prevalence of mental disorders and limited availability of specialised mental health care, particularly in rural areas. Participating general practitioners reported valuing the programme’s flexible implementation and coherent structure, which they found to be aligned with their professional needs. They further noted that the programme reduced common barriers to participation and supported engagement despite the presence of high workloads. Participants primarily emphasised increased confidence, which was associated with self-reported adjustments in their everyday clinical practice. These reflections are representative of the participants’ reported experiences and must be interpreted more cautiously, as they may indicate their motivation to participate rather than actual impacts on engagement and practice adjustments.

This qualitative study provides initial insights into the perceptions and experiences of the participants regarding the programme, enabling a better understanding of the recurrent patterns observed in their accounts and the contextual factors and experiences that shape them.

Consistent with previous studies on ECHO programmes [18,19,20,21], participants reported generally positive perceptions of the format and structure of the “ECHO School of Psychiatry”, noting the regular remote sessions as useful and supporting engagement from any location. Case-based discussions were regarded as a valuable component, corroborating findings from other studies [20,21]. Participants identified the guidance and support, as well as the safe learning environment, provided by the psychiatry experts as key strengths of the programme. These findings align with the extant literature suggesting that the non-hierarchical nature of the ECHO telementoring model is essential for effective knowledge transfer [19].

Participants’ reflections on the programme’s suitability, convenience, and thematic relevance empirically support the implementation literature by Serhal et al. [22]. They demonstrate that alignment with the core principles of ECHO, including session structure, combined with contextual and audience-specific adjustments, underpins the practical utility and positive evaluation of the programme. Furthermore, it provides support for healthcare professionals facing current challenges [23]. Notably, these insights are based on participants’ perceptions and may be influenced by context-specific factors, such as the Latvian healthcare system and professional cultural norms. Understanding and addressing the needs of the audience, including aligning topics with participants’ requirements, is highlighted by Allison et al. as a key factor for the successful design and implementation of ECHO programmes [24]. However, it is crucial to interpret these findings with a degree of caution, as they reflect self-reported experiences from a small, voluntary sample and may be influenced by social desirability bias, which is acknowledged as a study limitation.

Within this sample, doctors did not report any barriers to participation, which contrasts with previous studies where frequency, timing and length of the sessions are commonly reported as obstacles [21,25,26,27,28]. This finding may reflect the programme’s perceived flexibility; however, it is essential to note that participants were asked in advance about the timing of sessions that would be convenient for them. Consequently, this finding may be indicative of characteristics inherent to the motivated study sample and the voluntary response sampling method employed rather than an inherent advantage of the programme. Additionally, it may indicate the underrepresentation of less satisfied doctors’ perspectives, which is a limitation acknowledged in this study.

Participants perceived that engagement in the “ECHO School of Psychiatry” increased their confidence in clinical practice and reported making some adjustments in patient assessment and management, including communication and addressing of mental health-related concerns. This finding is consistent with the existing literature on ECHO programmes [16,18,19,27,28,29,30,31,32]. Importantly, participants reported more frequent use of screening instruments, primarily PHQ-9, following participation. This tool has been validated for the Latvian population and is recommended [33] as effective for depression screening in primary care. It should be noted that screening instruments can support clinical assessment but do not replace formal diagnosis according to ICD-10 criteria. These self-reported changes may suggest perceived improvements in timely recognition and provision of care, which is particularly relevant considering the underdiagnosis of depression in primary care both globally [34] and in Latvia [8,35].

Consistent with previous research, the findings point to participants viewing the ECHO model-based initiatives as a potentially useful approach for disseminating knowledge and engaging professionals in rural and underserved settings [20,25,36], by linking primary care providers with an academic psychiatry expert team.

Participants regarded the ECHO programme as a flexible tool that could support the delivery of mental health care, an aspect essential in primary care [36]. Furthermore, this study adds to the literature on the perceived usefulness of the ECHO model, illustrating its value not only as a disease-focused educational initiative but also as a broader framework for providing focused training within a specific field [21,28,29,37].

Participants’ reflections are consistent with the objectives of the ECHO model, indicating that it may serve as a supportive approach for continuing professional development [38]. Participants noted that the programme contributed to their competencies and capacity, thereby supporting more timely patient management.

The findings align with the educational theoretical basis of the ECHO model as described by Arora et al. [39], which incorporates social cognitive theory and situated learning theory to support participants in acquiring knowledge and applying it to modify their practice. Based on participants’ reflections, the “ECHO School of Psychiatry” appeared to partially reflect the principles of community of practice theory by creating a learning environment composed of specialists facing similar challenges. Nevertheless, limited peer interaction suggests that the programme did not completely realise all aspects of these theoretical principles.

The study highlights participants’ perceptions of the programme’s relevance and draws attention to some broader challenges within the primary care system in Latvia, emphasising the critical role of general practitioners as the first point of contact for patients with mental health concerns or disorders [40,41]. The participants’ views suggest that the ECHO model could offer a useful framework for addressing certain aspects of these challenges.

In line with previous research, participants suggested strategies to enhance engagement among ECHO learners [24]. This reflects the key perceived limitation of the “ECHO School of Psychiatry”: its limited capacity to address professional isolation. This finding contrasts with several prior studies that identified fostering collaboration and creating a sense of community as a core strength of the programme [28,29]. These results should be interpreted in the context of Latvia’s cultural and healthcare system characteristics. The healthcare system is relatively centralised, with most specialists concentrated in major cities, contributing to a shortage of physicians in regional areas. While this shortage may increase the perceived value of continuing educational programmes and encourage participation in such initiatives, several contextual factors may limit engagement. These include the unfamiliar structure of the telementoring format, cultural norms such as adherence to traditional professional hierarchies and cautious attitudes towards new collaborative models, as well as varying levels of technological access and proficiency, which together may reduce active participation in discussions and limit engagement in such programmes overall. This primary limitation underscores the need for interdisciplinary engagement in structured educational initiatives, which has been recognised as a significant factor influencing the transformative potential of ECHO model programmes to improve the delivery of patient care [29,42].

Extending from this limitation, participants suggested considering expansion of the programme beyond general practitioners to reach a broader audience of physicians, emphasising the potential value of an interdisciplinary educational environment to foster collaboration and optimise care. This observation highlights a broader challenge: ECHO initiatives alone are unlikely to transform the traditionally narrow, discipline-specific approach to patient management.

This qualitative study has several limitations. The use of a voluntary sampling method may have resulted in the overrepresentation of more motivated and engaged participants, while the perspectives of potentially dissatisfied general practitioners may be underrepresented. Additionally, information on reasons for non-participation was not systematically collected, and therefore barriers to engagement experienced by those who did not participate may not have been fully captured.

Furthermore, the study sample consisted of only women general practitioners, reflecting both the gender distribution typical of the general practitioner population in Latvia [43] and the demographic profile of programme participants. This should be considered when interpreting the findings and may limit transferability to more gender-diverse contexts.

Another potential limitation relates to the involvement of M.B. in conducting the interviews due to prior contact with the participants. Although M.B. was not involved in teaching or evaluating the participants and had no hierarchical role within the programme, she was involved in programme coordination, which could be acknowledged by the participants as affiliation with the programme’s management. This represents a possible bias due to the interviewer–participant relationship, which might have influenced participants’ willingness to provide more critical feedback and may have contributed to social desirability bias that cannot be fully excluded. This potential bias must be considered when interpreting the predominantly positive findings. To support reflexivity and enhance analytic rigour, all stages of thematic analysis were conducted collaboratively within the research team.

Furthermore, the relatively short duration of the interviews (an average of 14 min) represents a further methodological limitation of this study. Although semi-structured interviews were designed to explore participants’ experiences using a focused topic guide, time constraints may have limited the depth of the data collected and the ability to explore more complex themes. This may have reduced the opportunity to capture more nuanced perspectives and complex aspects of participants’ experiences. The relatively short duration of the interviews was likely influenced by participants’ busy clinical schedules and limited availability; however, it is important to acknowledge that this may have constrained the completeness, depth, and overall scope of the findings. Consequently, relatively short interviews could limit the richness of the data obtained and the extent to which full thematic saturation was achieved, despite the fact that consistent patterns in participants’ accounts were identified through thematic analysis. This should be considered when interpreting the findings, as additional or more in-depth interviews could have yielded further nuances and perspectives.

The predominantly positive tone of participants’ accounts therefore requires more cautious interpretation. While the accounts may reflect genuine experiences, they cannot be fully disentangled from the voluntary nature of participation, the participants’ perception of the interviewer’s affiliation with the programme, or potential social desirability effects. Efforts were made to identify and discuss any data that might alter the emerging themes, ensuring that alternative perspectives were considered. Despite collaborative analysis and reflective measures, complete analytic independence could not be assumed in the insider context of this research.

Furthermore, the small nature of the programme may limit the broader relevance and transferability of the findings. Additionally, the findings are based on participants’ subjective perceptions of the programme and its impact on their practice, which does not reflect objective changes in patient care and management. As the “ECHO School of Psychiatry” is the first ECHO-based telementoring initiative in Latvia, further evaluation will be necessary as the project expands, enabling a more comprehensive assessment of the model’s suitability and effectiveness for primary care providers.

The findings may be most transferable to similar voluntary continuing medical education programmes, particularly telementoring interventions in comparable healthcare systems and professional contexts.

A potential direction for future research could include the exploration of patient-level outcomes linked to general practitioners’ engagement in the ECHO programme. Further studies could investigate the long-term effects of participation on clinical practice and the sustainability of reported changes. Future research could employ longitudinal or mixed-methods designs and integrate objective clinical outcomes, which would provide a more comprehensive assessment of the programme’s impact. Combining subjective and measurable data would enable robust triangulation and offer stronger evidence regarding the sustainability and practical impact of practice changes resulting from participation. Additionally, the adaptation of the ECHO model to other medical professionals could be examined, with subsequent evaluation of its perceived effectiveness across diverse clinical contexts within the Latvian healthcare system. Methodologically, future studies could implement purposive sampling strategies, including less engaged participants and non-completers, to reduce potential self-selection bias.

## 5. Conclusions

This study provides initial insights into general practitioners’ perceptions of Latvia’s first telementoring initiative, the “ECHO School of Psychiatry”, identifying both its strengths and areas for improvement. The findings reflect participants’ views and experiences of the programme. They also acknowledge ongoing challenges in primary care and the need for further educational support, with the ECHO model being perceived by participants as a potential tool for sharing knowledge and learning, particularly in rural and underserved settings. Although the programme was perceived positively, these perceptions should be interpreted with caution, as they may be influenced by the voluntary nature of participation and social desirability bias, and may not capture the perspectives of all eligible participants.

## Figures and Tables

**Table 1:** Thematic structure of the “ECHO School of Psychiatry” telementoring programme.

Session Topic
Recurrent depressive disorder Clinical pathways and algorithms for depression Suicide risk assessment and prevention/Depression in bipolar affective disorder Depression in the context of substance use disorder Perinatal mental disorders/Affective and anxiety symptoms in patients with somatic illnesses Neurotic disorders Somatoform disorders Psychopharmacology

**Table 2:** Final interview topic guide.

Main Open-Ended Questions
What are your overall impressions of the “ECHO School of Psychiatry” programme? Which aspects of the programme did you find most positive, and why? Which aspects of the programme did you find less favourable, and why? What was the impact of the programme on your clinical practice and patient management? What factors limited your participation in the programme? How, in your opinion, could the programme be further improved? Are there any additional aspects of the programme that you consider essential that were not previously discussed?
**Follow-up prompts**
Timing and duration of seminars Structure and organisation of seminars Comprehensiveness and relevance of topics covered Case-based discussions Additional project materials provided by email Seminar recordings

**Table 3:** Themes and subthemes emerging from the data.

Themes	Subthemes
1. Participants’ perceptions of the structure and educational value of the programme	Suitability of the telementoring format and programme structure Relevance of the thematic scope Enhanced learning through guidance by psychiatry experts
2. Perceived impact of the programme on clinical practice and decision-making	Increased confidence Clarification of professional boundaries Changes in patient management approaches
3. Programme limitations in addressing professional isolation and fostering collaboration	Limited engagement of participants Technology as a mediator in communication
4. Suggestions for programme improvement	Extension of the thematic scope Expansion of the ECHO model to other medical fields Inclusion of supplementary materials and information Encouragement of active learning Enhancement of participant engagement Repetition of the programme

## Data Availability

The data of the study are available upon request from the corresponding author.
